# Supplementary material for: 5-Fluorouracil and irinotecan (SN-38) have limited impact on colon microbial functionality and composition in vitro
Source: PeerJ. 2017 Nov 16;5:e4017. doi: 10.7717/peerj.4017 (PMC5694655; doi:10.7717/peerj.4017)
Supplement: Supplemental Information 1 [file peerj-05-4017-s001.docx]

***Supplementary information***

**5-Fluorouracil and irinotecan (SN-38) have limited impact on colon microbial functionality and composition *in vitro***

Eline Vanlancker^1^, Barbara Vanhoecke^1^, Andrea Stringer^2^, Tom Van de Wiele^1#^

^1^ Center for Microbial Ecology and Technology (CMET), Ghent University, Ghent, Belgium.

^2^ Sansom Institute for Health Research, University of South Australia, Adelaide, Australia

#: Corresponding author:

Tom Van de Wiele, Center for Microbial Ecology and Technology (CMET), Coupure Links 653 Building A, 9000 Ghent, Belgium. Phone: +32 9 264 59 12, Fax: +32 9 264 62 48; Email: tom.vandewiele@ugent.be

Table of Contents

[Supplementary tables S3](#_Toc493681786)

[Supplementary figures S4](#_Toc493681787)

[Supplementary M&M S15](#_Toc493681788)

[References S16](#_Toc493681789)

# Supplementary tables

Table S1: Abundance of major phyla (%), based on Illumina Miseq sequencing of the 16S rRNA gene, for different types of samples in the M-SHIME runs with 5-FU (AV ±STDEV)

|  |  |  | Firmicutes | Bacteroidetes | Proteobacteria |
| --- | --- | --- | --- | --- | --- |
| Lumen | Day 0 | Control | 39,1 ± 31,9 | 23,6 ± 12,4 | 29,6 ± 22,0 |
|  |  | 5-FU | 41,8 ± 23,3 | 26,9 ± 14,1 | 26,4 ± 19,0 |
|  | Day 6 | Control | 28,5 ± 23,7 | 31,2 ± 15,3 | 30,3 ± 17,3 |
|  |  | 5-FU | 27,7 ± 19,6 | 46,1 ± 12,3 | 17,6 ± 9,9 |
| Mucus | Day 0 | Control | 67,0 ± 20,8 | 11,3 ± 9,5 | 15,2 ± 15,4 |
|  |  | 5-FU | 71,3 ± 13,2 | 11,1 ± 6,3 | 12,6 ± 7,9 |
|  | Day 6 | Control | 48,1 ± 16,8 | 16,5 ± 11,3 | 18,9 ± 11,5 |
|  |  | 5-FU | 47,9 ± 12,8 | 21,7 ± 8,8 | 16,7± 9,3 |

Table S2: Abundance of major phyla (%), based on Illumina Miseq sequencing of the 16S rRNA gene, for different types of samples in the M-SHIME runs with SN-38 (AV ±STDEV)

|  |  |  | Firmicutes | Bacteroidetes | Proteobacteria |
| --- | --- | --- | --- | --- | --- |
| Lumen | Day 0 | Control | 46,9 ± 15,7 | 23,2 ± 14,7 | 27,0 ± 16,4 |
|  |  | SN-38 | 36,5 ± 18,2 | 33,2 ± 9,3 | 27,0 ± 11,9 |
|  | Day 6 | Control | 23,1 ± 9,0 | 53,3 ± 7,6 | 23,2 ± 10,9 |
|  |  | SN-38 | 17,8 ± 8,1 | 51,2 ± 5,9 | 24,6 ± 9,6 |
| Mucus | Day 0 | Control | 75,4 ± 19,9 | 10,7 ± 8,2 | 11,3 ± 16,8 |
|  |  | SN-38 | 73,0 ± 17,8 | 14,3 ± 7,8 | 10,1 ± 12,6 |
|  | Day 6 | Control | 44,8 ± 15,8 | 19,3 ± 2,9 | 23,5 ± 9,1 |
|  |  | SN-38 | 44,2 ± 4,6 | 21,6 ± 4,4 | 21,4 ± 6,5 |

# Supplementary figures


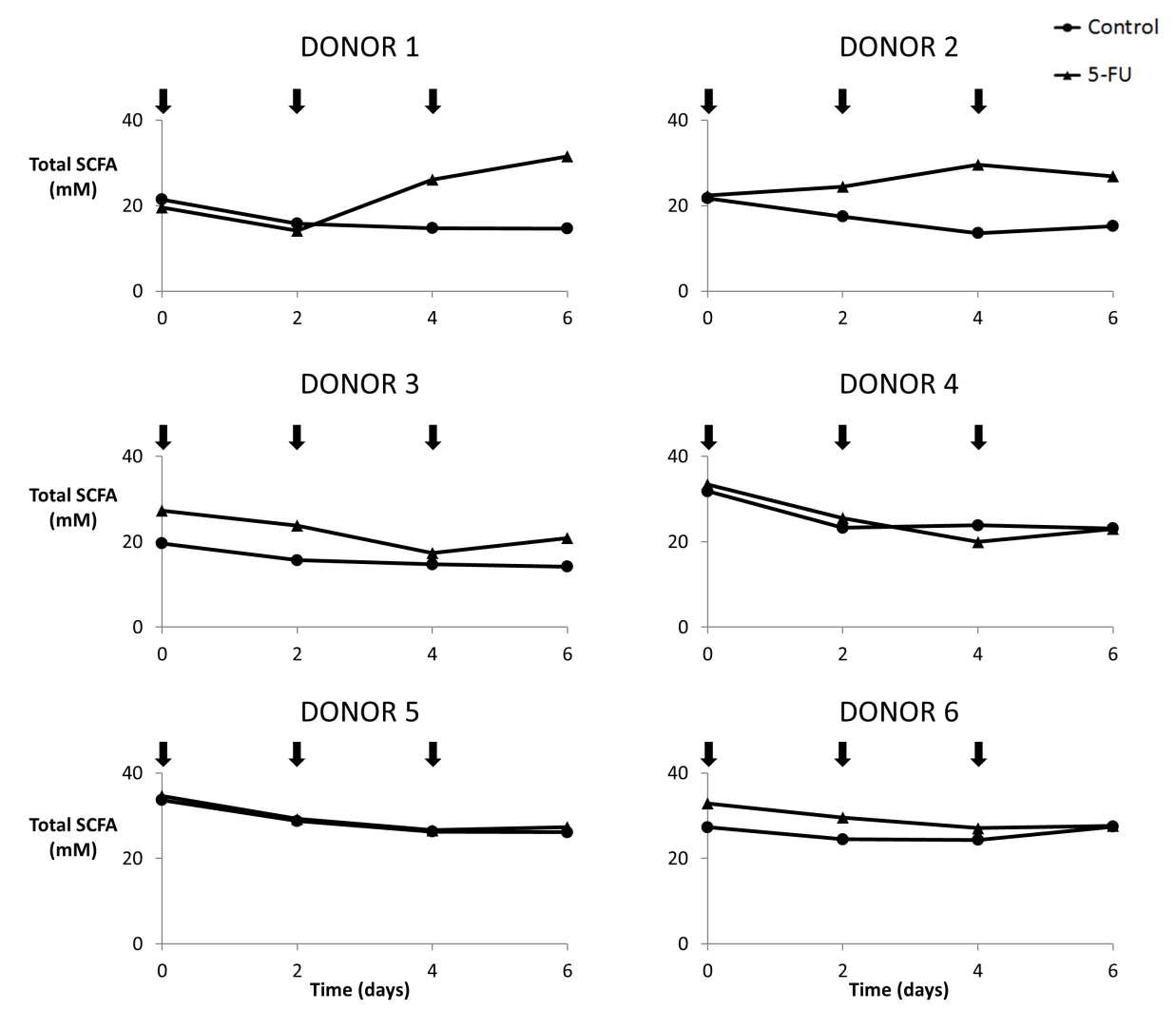


Figure S1: At 10 µM in the luminal part of the M-SHIME, 5-FU has limited effect on mucosal total short chain fatty acid concentrations (arrows indicate the time of dosing)


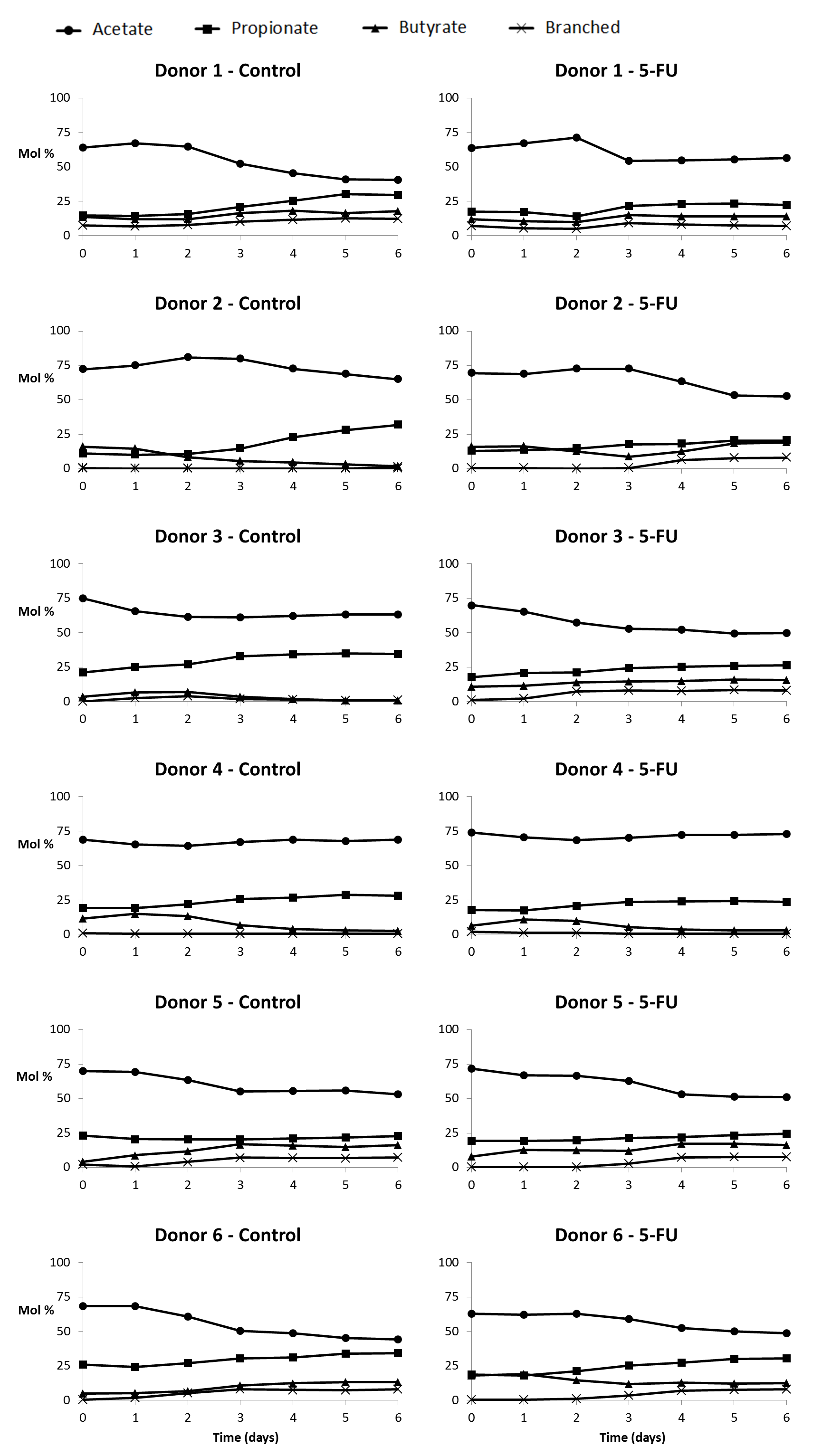


Figure S2: At 10 µM in the luminal environment of the M-SHIME, 5-FU has no effect on luminal relative short chain fatty acid concentrations


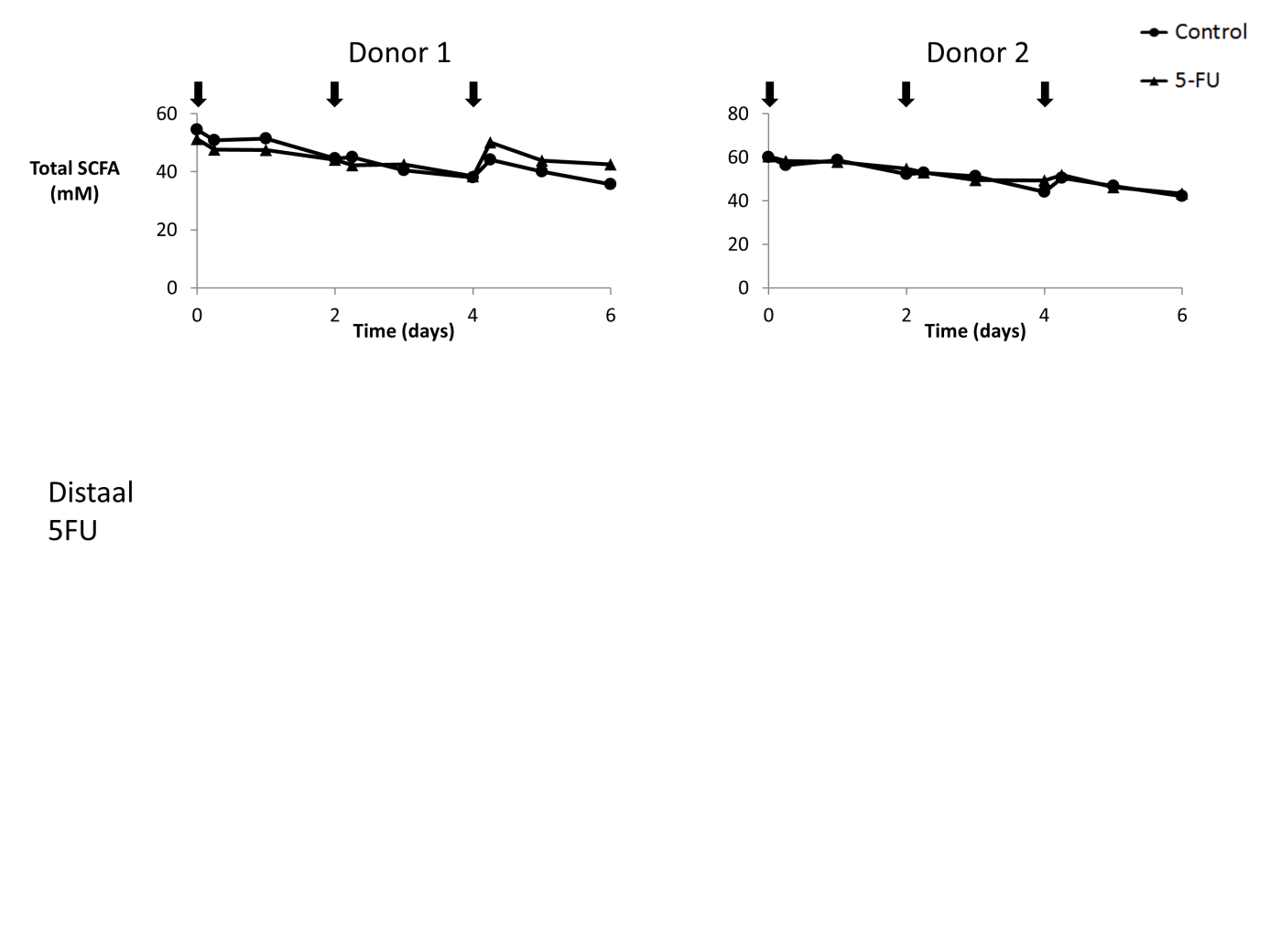


Figure S3: At 10 µM in the luminal environment of the distal part of the M-SHIME, 5-FU has no effect on luminal relative short chain fatty acid concentrations


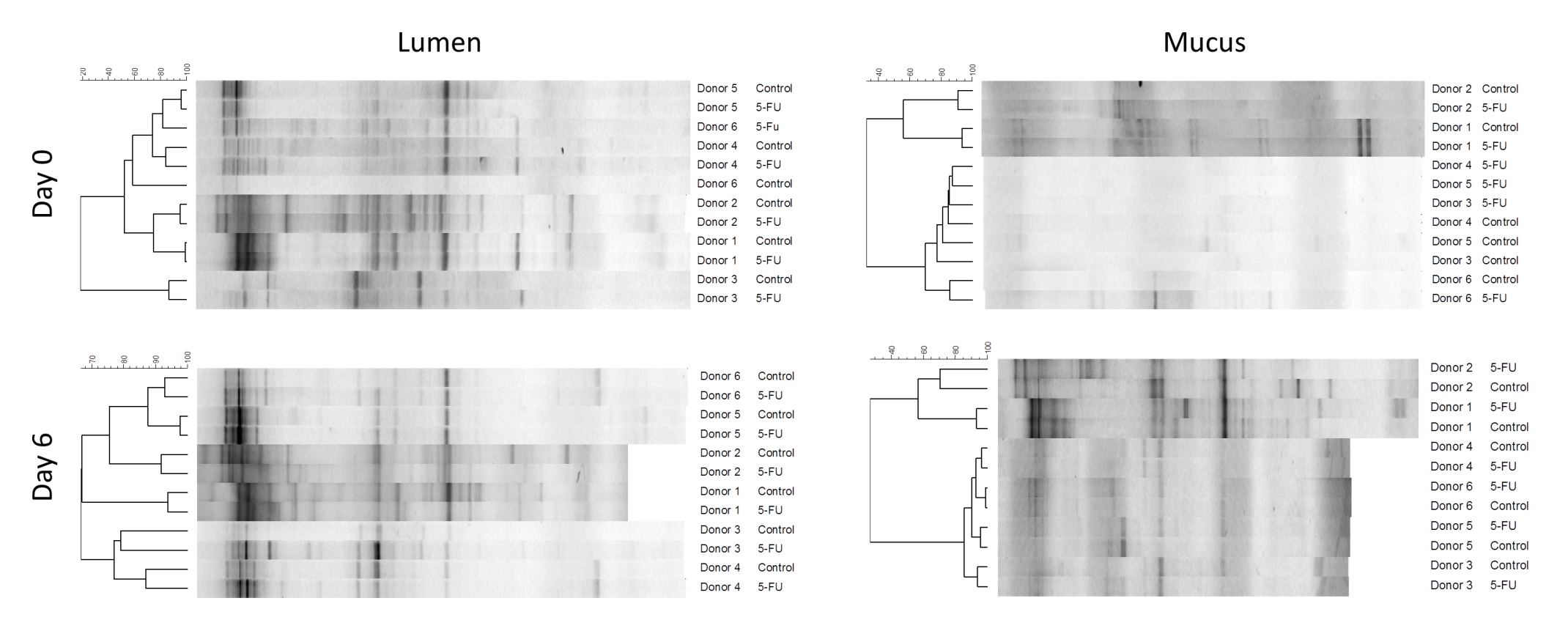


Figure S4: At 10 µM in the mucosal part of the M-SHIME, 5-FU has no clear effect on both the luminal and the mucosal microbial composition as shown with DGGE

Figure S5: Relative abundances of the 20 most abundant genera of the microbial community of the M-SHIME run with 5-FU based on Illumina Miseq sequencing of the 16S rRNA gene


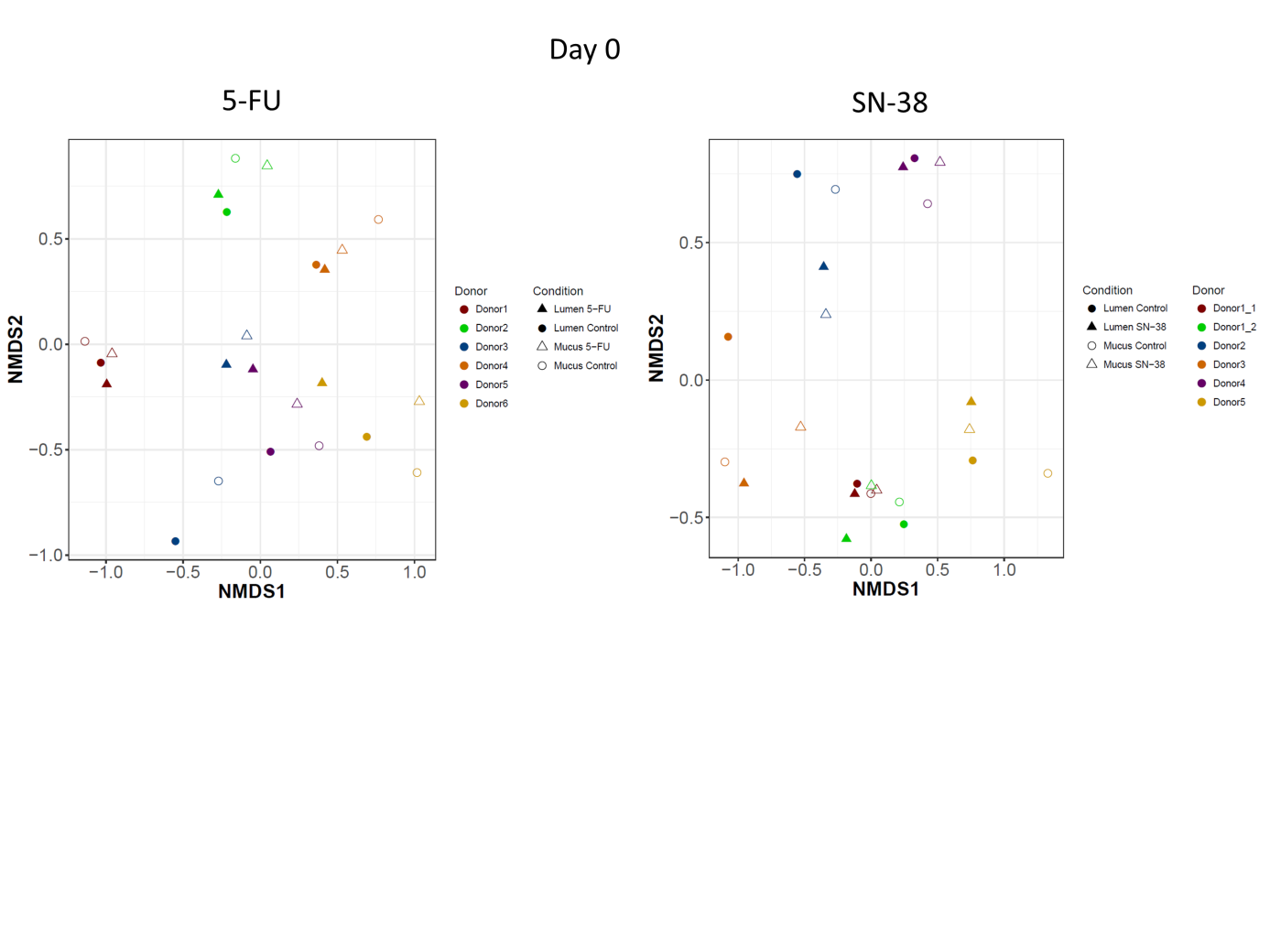


Figure S6: NMDS plots based on Bray Curtis dissimilarities of Illumina data on day 0 showed high interindividual variability


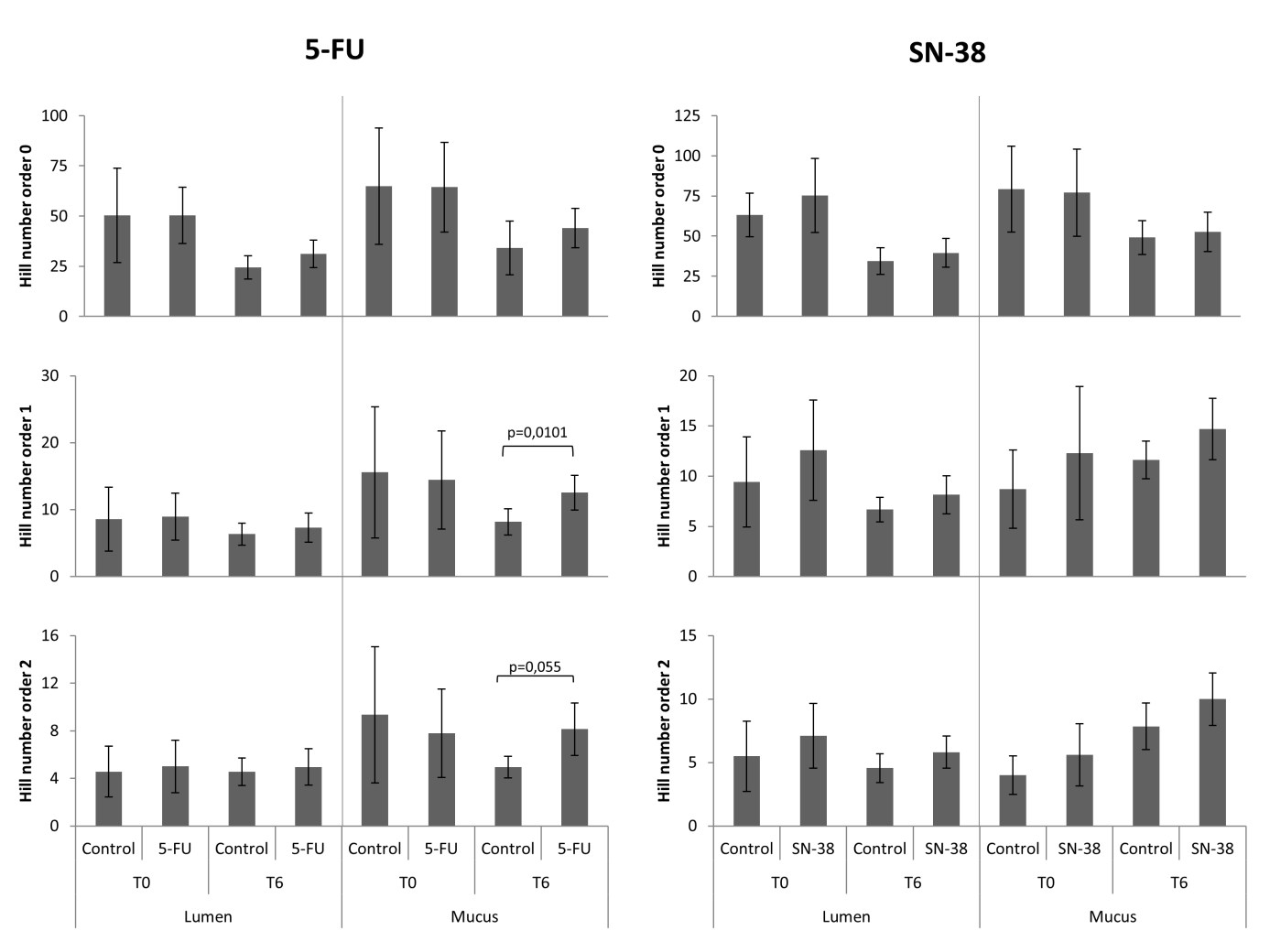


Figure S7: Hill numbers based on Illumina sequencing of the 16S rRNA gene as a measure for diversity. Order 0 displays species richness, order 1 the exponential of Shannon’s entropy index and order 2 the inverse of Simpson’s concentration index.


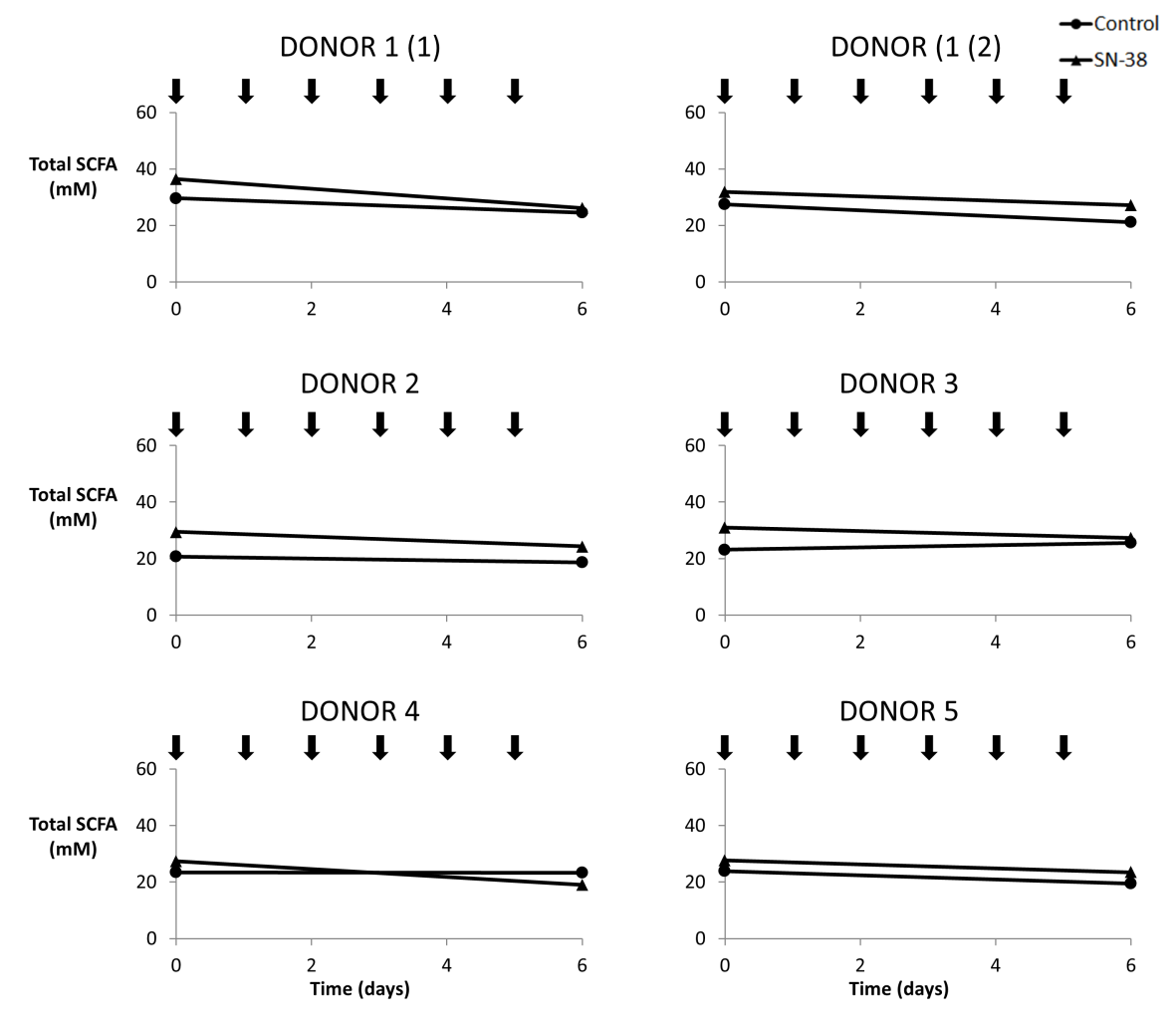


Figure S8: At 10 µM in the luminal part of the M-SHIME, irinotecan (SN-38) has no effect on mucosal total short chain fatty acid concentrations (arrows indicate the time of dosing)


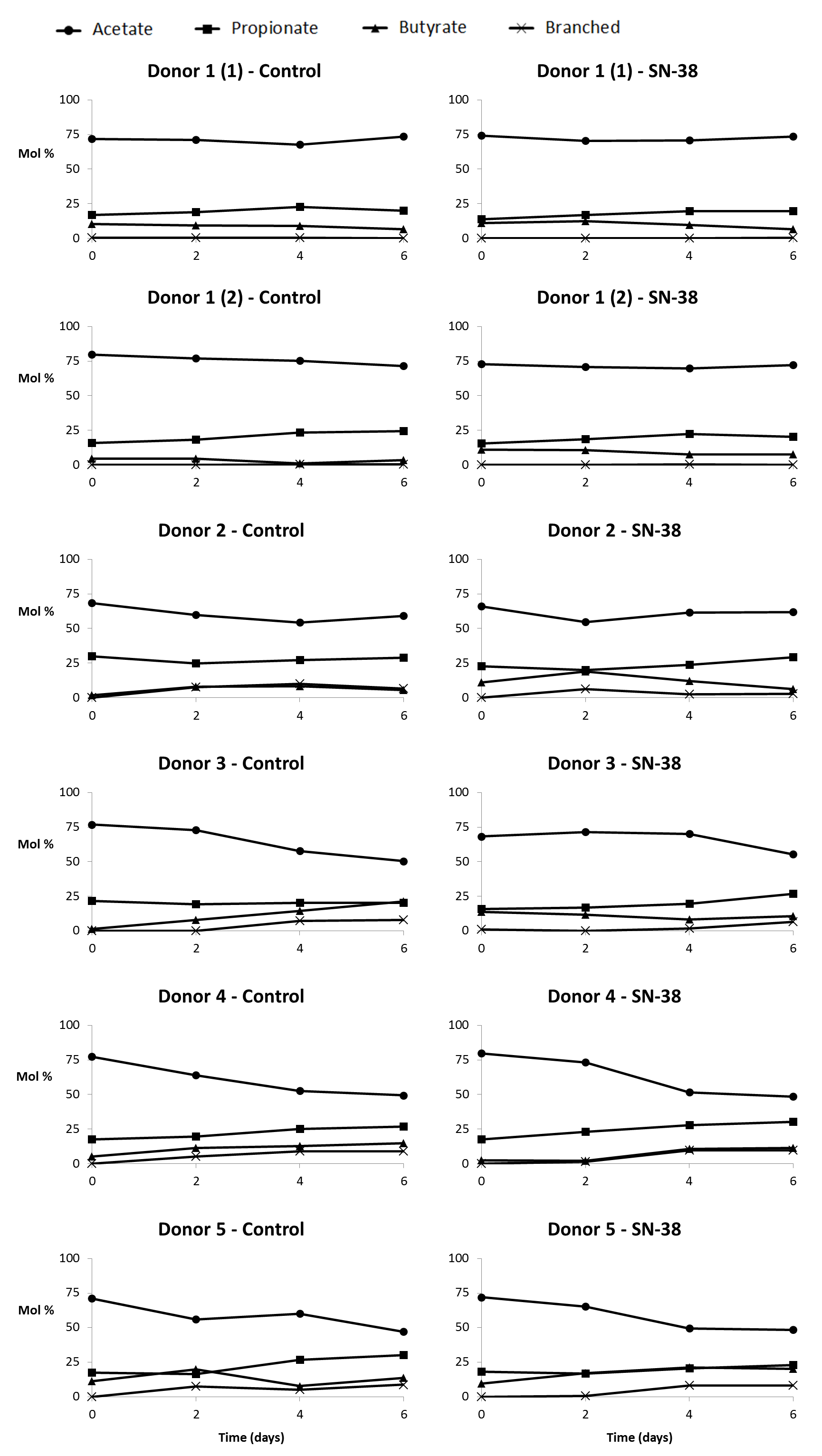


Figure S9: At 10 µM in the luminal part of the M-SHIME, irinotecan (SN-38) has no effect on luminal relative short chain fatty acid concentrations


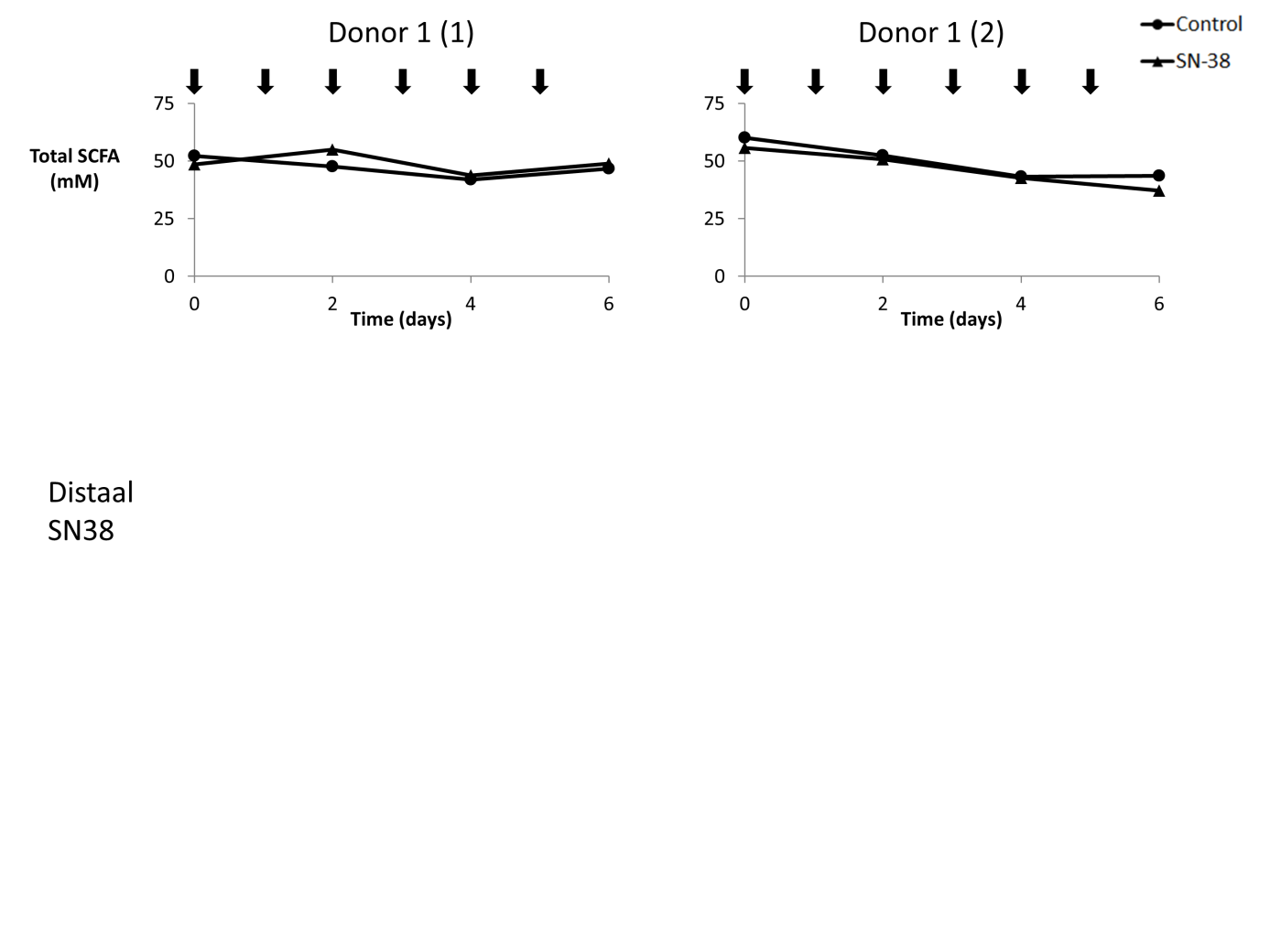


Figure S10: At 10 µM in the luminal part of the distal environment of the M-SHIME, irinotecan (SN-38) has no effect on luminal total short chain fatty acid concentrations


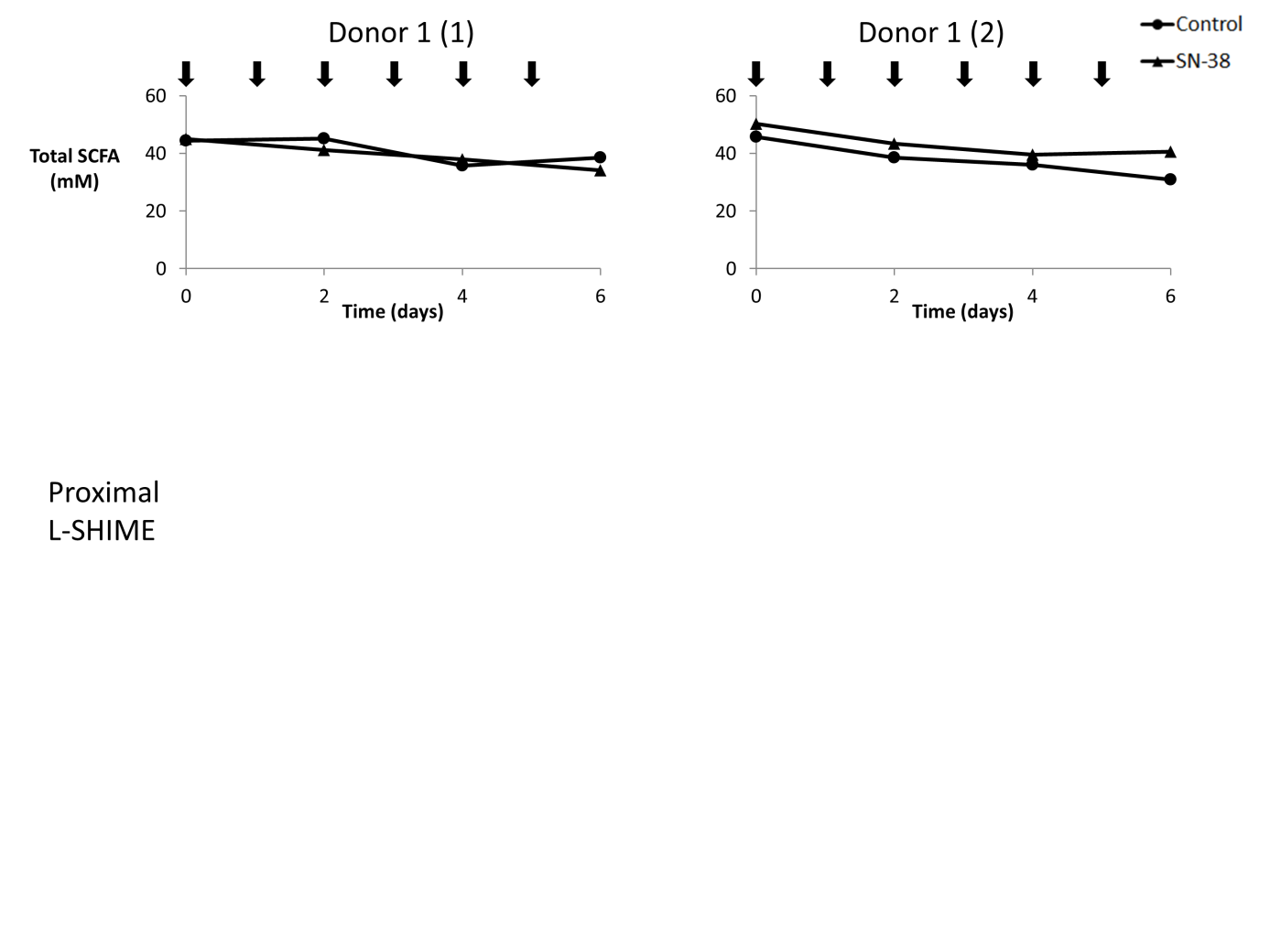


Figure S11: At 10 µM in the luminal part of the proximal environment of the L-SHIME, irinotecan (SN-38) has no effect on luminal total short chain fatty acid concentrations


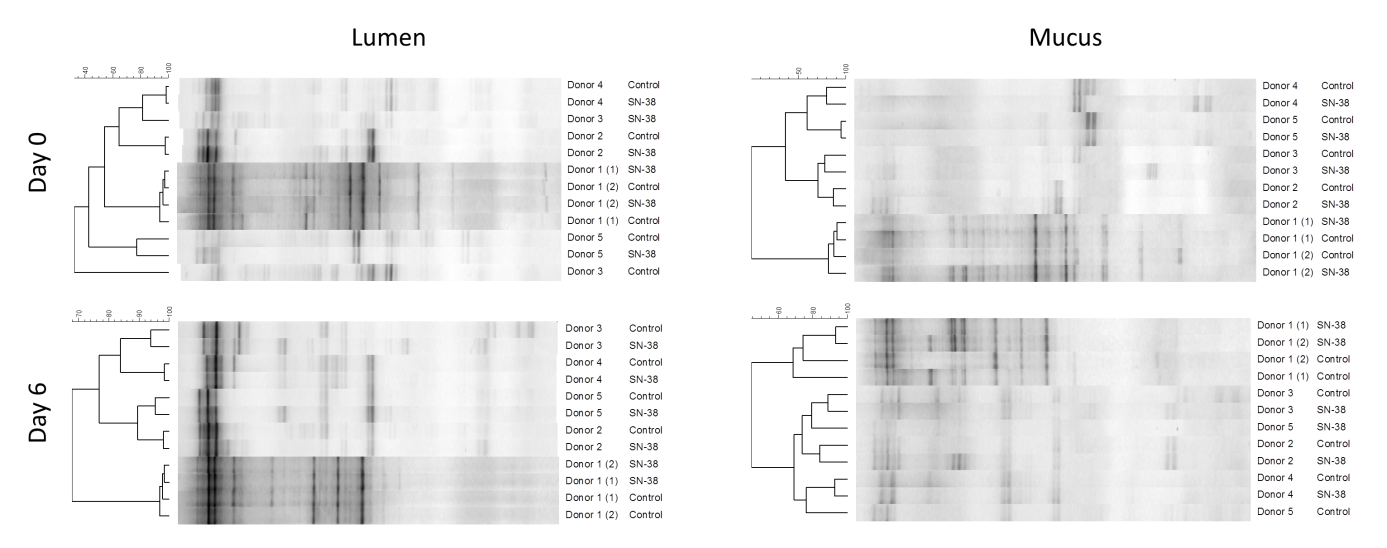


Figure S12: At 10 µM in the luminal part of the SHIME, SN-38 has no clear effect on both the luminal and the mucosal microbial composition as shown with DGGE

Figure S13: Relative abundances of the 20 most abundant genera of the microbial community of the M-SHIME run with SN-38 based on Illumina Miseq sequencing of the 16S rRNA gene

# Supplementary M&M

**Denaturing Gradient Gel Electrophoresis (DGGE)**

The 16S rRNA gene region was amplified by means of PCR using the PRBA338F-GC and 518R primers targeting the V3 region (Muyzer et al. 1993; Ovreas et al. 1997). The PCR program consisted of 10 min 95°C; 30 cycles of 1 min 94°C, 1 min of 53°C, 2 min of 72°C; and a final elongation for 10 min at 72°C. Amplification products were analysed by gel electrophoresis (1.2 % (w/v) agarose).

The DGGE was performed using the INGENYphorU System (Ingeny International BV, The Netherlands), based on the protocol Muyzer et al. (1993). The PCR fragments were mixed with loading buffer (5:1) before loading onto 8 % (w/v) polyacrylamide gels with denaturing gradients ranging from 45 % to 60 % (where 100 % denaturant contains 7 M urea and 40 % formamide). The electrophoresis was run for 16 hours at 60 °C and 120 V. Staining and analysis of the gels was performed as described previously (Boon et al. 2000). To process and compare the different gels, a homemade marker of different PCR fragments was loaded on each gel (Boon et al. 2002). The normalization and analysis of the DGGE gel patterns was carried out with the BioNumerics software 5.10 (Applied Maths, Sint-Martens-Latem, Belgium). The calculation of the similarity matrix was based on the Pearson correlation coefficient, and clustering was performed with the unweighted pair-group average method algorithm (UPMA).

# References

Boon N, De Windt W, Verstraete W, and Top EM. 2002. Evaluation of nested PCR-DGGE (denaturing gradient gel electrophoresis) with group-specific 16S rRNA primers for the analysis of bacterial communities from different wastewater treatment plants. *Fems Microbiology Ecology* 39:101-112. 10.1111/j.1574-6941.2002.tb00911.x

Boon N, Goris J, De Vos P, Verstraete W, and Top EM. 2000. Bioaugmentation of activated sludge by an indigenous 3-chloroaniline-degrading Comamonas testosteroni strain, I2gfp. *Applied and Environmental Microbiology* 66:2906-2913. 10.1128/aem.66.7.2906-2913.2000

Muyzer G, Dewaal EC, and Uitterlinden AG. 1993. Profiling of complex microbial-populations by denaturing gradient gel-electrophoresis analysis of polymerase chain reaction-amplified genes-coding for 16S ribosomal-RNA. *Applied and Environmental Microbiology* 59:695-700.

Ovreas L, Forney L, Daae FL, and Torsvik V. 1997. Distribution of bacterioplankton in meromictic Lake Saelenvannet, as determined by denaturing gradient gel electrophoresis of PCR-amplified gene fragments coding for 16S rRNA. *Applied and Environmental Microbiology* 63:3367-3373.
